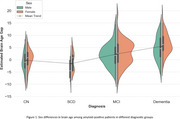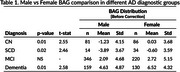# Sex Differences in Brain Age Gap Estimation Across Alzheimer's Disease Diagnostic Groups

**DOI:** 10.1002/alz70862_110227

**Published:** 2025-12-23

**Authors:** Reza Rajabli, Mahdie Soltaninejad, D Louis Collins

**Affiliations:** ^1^ McConnell Brain Imaging Centre, Montreal Neurological Institute, McGill University, Montreal, QC Canada

## Abstract

**Background:**

Brain Age Gap (BAG), the difference between age estimated from brain MRI and chronological age, is a potential feature for quantifying an individual's overall level of neurodegeneration. As a global measure, BAG can be used to examine differences in the extent of neurodegeneration across various groups. In this study, we apply BAG to amyloid positive subjects and investigate potential sex differences in different diagnostic groups.

**Method:**

We trained five different models on UK Biobank (UKBB) and the Mayo Clinic Study of Aging (MCSA) data, with an age range of [45, 89] and nearly balanced sex distribution, to build an ensemble model for predicting brain age from T1w images. To ensure the model's generalization within the training age range, we used robust image preprocessing methods, massive data augmentation, and model regularization techniques (Rajabli 2024). Using our brain age prediction model, without further fine‐tuning, we estimated BAG on Alzheimer's Disease Neuroimaging Initiative (ADNI) samples.

**Result:**

We estimated the brain age gap for all cognitively normal subjects, regardless of amyloid status, and found no significant sex difference (‐0.24 ± 3.85 for males, ‐0.05 ± 4.04 for females), indicating that our model is not biased toward either sex. As shown in previous studies and reaffirmed by our model, BAG increases along the AD trajectory (Figure 1). After correcting for age and the ADAS13 cognitive score, we found a residual statistically significant sex difference (*p* < 0.05) in BAG estimations, with amyloid‐positive female brains appearing older than their male counterparts across all diagnostic groups except MCI, as determined by an ANCOVA test. Table 1 summarizes the statistical analysis.

**Conclusion:**

We showed that female brains appear older than male brains in most diagnostic groups. While we corrected for age and ADAS13 within each group, this finding may suggest that females have greater cognitive reserve than males.